# Effectiveness of a guided internet-based intervention for procrastination among university students – A randomized controlled trial study protocol

**DOI:** 10.1016/j.invent.2023.100612

**Published:** 2023-03-03

**Authors:** Arpana Amarnath, Sevin Ozmen, Sascha Y. Struijs, Leonore de Wit, Pim Cuijpers

**Affiliations:** a Department of Clinical, Neuro and Developmental Psychology, Amsterdam Public Health Research Institute, Vrije Universiteit Amsterdam, the Netherlands; b WHO Collaborating Centre for Research and Dissemination of Psychological Interventions; c Institute of Clinical Psychology, Leiden University, Leiden, the Netherlands

**Keywords:** Internet-based intervention, Guided intervention, Procrastination, University students, Mental heath

## Abstract

*Procrastination* is a widespread problem that is highly prevalent among the young adult population and is associated with several negative consequences. However, current evidence on the effectiveness of e-health interventions for procrastination either lack a comparison to an inactive control, do not include a student population or are of poor quality. This protocol describes the design of a trial that will overcome these limitations and examine the effectiveness of a guided internet-based intervention (*GetStarted*) to reduce problematic procrastinating behaviors in college students compared to a waitlist control. This study will be a two-armed randomized controlled trial with a calculated sample size of *N* = 176. Participants will be students from seven universities in the Netherlands. The intervention group will receive a four-week e-coach-guided intervention for procrastination. The waitlist control group will get access to treatment four weeks after randomization. Assessments will take place at baseline, post-test (4 weeks post-baseline) and follow-up (6 months post-baseline). Data will be analyzed with an intent-to-treat principle. The primary outcome is change in procrastination behaviors measured on the Irrational Procrastination scale (IPS). Secondary outcomes are depression, anxiety, stress, and quality of life. Additionally, sociodemographic characteristics of the participants, satisfaction with treatment, program usability, satisfaction with e-coach and treatment adherence will be examined as potential moderators. The results from this study can build evidence for the effectiveness of a guided internet-based intervention for treating procrastination in college students. Should it be effective, *GetStarted* could provide a flexible, low-intense and cost-effective treatment for procrastination and prevent common mental health problems in college students.

**Trial registration:**

This trial is registered at ClinicalTrials.gov Protocol Registration and Results System (Trial number: NCT05478096).

## Highlights

• Many university students procrastinate, which can be associated with poor mental health, low academic achievements, and limited career opportunities.
• Guided online interventions have the promise to overcome several barriers associated with treating procrastination among university students.
• Evidence on the effectiveness of guided internet-based interventions for procrastination are scarce and of poor quality.
• High-quality trials are needed to provide the most appropriate intervention for university students at risk for the negative consequences of procrastination.

## 1 Introduction

Procrastination, which refers to the process of voluntarily yet irrationally delaying an intended or necessary course of action (Steel, 2007), is a widespread problem that is highly prevalent among the young adult population (Beutel et al., 2016). It was previously estimated that 20 % of the adult population self-identify as chronic procrastinators (Ferrari et al., 2005). This rate is a staggering 50 % for university students across many countries and continues to increase (Solomon and Rothblum, 1984; Tice and Baumeister, 1997; Pychyl et al., 2000; Schouwenburg et al., 2004; Steel, 2007; Ozer et al., 2009). Chronic procrastination is associated with several negative consequences such as low self-esteem, excessive worrying, anger problems, shame, increased stress, depression, anxiety, and overall lower satisfaction with life (Sirois, 2007; Flett et al., 2016; Stead et al., 2010; Beutel et al., 2016). Apart from the negative psychological consequences, students who procrastinate have lower grade point averages (GPAs), assignment scores, and examination scores when compared to those who do not (Tice and Baumeister, 1997; Steel et al., 2001; Kim and Seo, 2015). Subsequently, they experience adverse long-term consequences such as poor general health, problems in social relationships, financial struggles and limited career opportunities (Patrzek et al., 2012; Höcker et al., 2017; Stead et al., 2010). This indicates that students, in particular, could experience more negative consequences with procrastination compared to the adult population (Steel and Klingsieck, 2016).

Previous studies have suggested that there are treatments available to target procrastination in students, with cognitive behavior therapy (CBT) being the most effective treatment (van Eerde and Klingsieck, 2018). However, several barriers to seeking and receiving treatment need to be accounted for to implement treatment successfully. These barriers include underestimating the problem, fear of stigmatization, perceived lack of time, low financial resources, preference for self-management and low awareness of available resources (Czyz et al., 2013; Eisenberg et al., 2007). E-health interventions, which can be accessed quickly, anonymously, and on a flexible schedule, could be a possible cost-effective solution to several of these barriers (Andersson et al., 2014; Ebert et al., 2017). Studies examining the effectiveness of e-health interventions, specifically targeting procrastination, show that e-health is effective in reducing procrastination (Eckert et al., 2018; Lukas and Berking, 2018). For example, a recent randomized controlled trial showed that chat-based counselling was as effective as face-to-face counselling for reducing procrastinating behaviors (Gieselmann and Pietrowsky, 2016). In line with this, Rozental et al. (2018a), found that unguided internet-based treatment was as effective as a group-based treatment for procrastination. While these studies provide strong evidence for the effectiveness of e-health treatments for procrastination, they lack an inactive comparator to be able to distinguish the impact of these interventions precisely. Additionally, previous studies that have examined comparisons to inactive controls either lack a comparison to an e-health intervention, do not include a student population or are of poor quality with low sample sizes and a high risk of bias, specifically regarding blinding of outcome assessors (Rozental et al., 2018b). This warrants the need for high quality and adequately powered trial to test the effectiveness of an e-health intervention compared to an inactive control for procrastination among students.

Another important potential barrier to effectively treating procrastination is treatment adherence. Rozental et al. (2015a) found that many individuals who receive treatment for procrastination struggle to adhere to and keep up with treatment. Guided treatments have previously been shown to result in better treatment outcomes (Cuijpers et al., 2019). However, unguided treatments are more scalable and affordable (Fairburn and Patel, 2017; Karyotaki et al., 2017). One possible solution is guidance by non-clinicians, which has the promise to be equally effective compared to clinician guidance (Leung et al., 2022). Clinical psychology students, in particular, have successfully supported face-to-face interventions for academic procrastination (Wang et al., 2017). Our study employs trained clinical psychology students as e-coaches to guide the treatment. Should this be an effective form of guidance, it overcomes existing barriers of limited resources in the form of limited available psychologists and counsellors (Watkins et al., 2012). Additionally, it makes treatment more affordable as psychology students offer e-health guidance for significantly lower costs compared to licensed specialists.

Therefore, the present study aims to evaluate the effectiveness of a guided internet-based intervention for procrastination (*GetStarted*) in university students in the Netherlands. Furthermore, since procrastination is associated with various other psychological outcomes (Sirois, 2007; Flett et al., 2016; Stead et al., 2010; Beutel et al., 2016), we will evaluate the effect of this treatment on secondary outcomes such as depression, anxiety, stress, and quality of life. Finally, we aim to gather evidence for potential moderators to treatment effectiveness. These will be socio-demographic characteristics of the participants, satisfaction with treatment and e-coach, system usability and treatment adherence. This will be done to identify target groups that could benefit most from treatment. The main research question is whether *GetStarted* is effective in reducing problematic procrastinating behaviors in university students compared to a waitlist control. The secondary research questions include: What are the effects of *GetStarted* on secondary outcomes of depression, anxiety, stress, and quality of life? Moreover, are there any moderators of treatment effectiveness?

## 2 Methods

### 2.1 Study design/setting

**Fig. 1:**
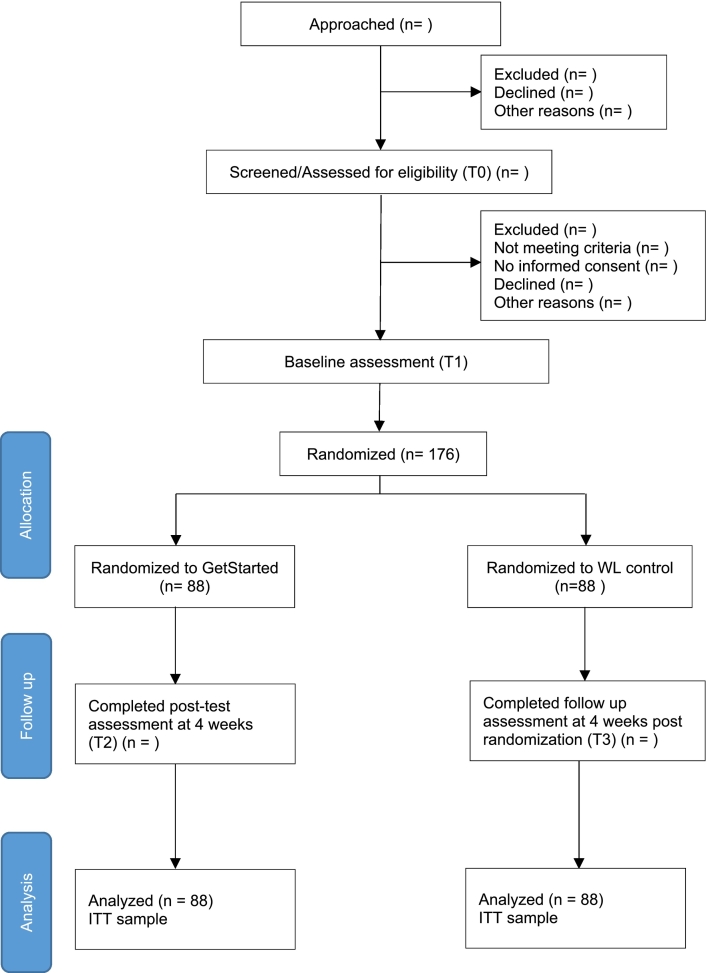
Participant flow diagram with targeted numbers.

This study is a two-armed randomized controlled trial comparing the effectiveness of a guided internet-based intervention program, *GetStarted*, to a waitlist control receiving no intervention for four weeks. A flow chart of the recruitment process and treatment period based on the recommendations of the CONSORT 2010 statement is shown in Fig. 1. This study is conducted within the Caring Universities project. Caring Universities is a consortium of 7 universities in the Netherlands. The project is a part of the World Health Organization (WHO) World Mental Health International College Student Initiative (WMH-ICS) (Cuijpers et al., 2019), aimed at improving our knowledge of university students' mental well-being and subsequently offering accessible web-based interventions to students in need.

The study protocol follows the recommendations of the SPIRIT 2013 Checklist for clinical trial protocols (Chan et al., 2013) and was approved by the Scientific and Ethical Review Board of all participating universities in the Netherlands: Vrije University Amsterdam, Leiden University, Maastricht University, and Utrecht University, Erasmus University, University of Amsterdam, Inholland University of Applied Sciences. The trial is registered on clinicaltrials.gov (NCT05478096) and is conducted and reported according to the CONSORT 2010 statement and the guidelines for executing and reporting research on internet-based interventions (Schulz et al., 2010; Proudfoot et al., 2011). The program (*GetStarted*) is administered in all the participating universities mentioned above.

### 2.2 Participants

Participants are domestic or international undergraduate, graduate or PhD students enrolled in the seven universities mentioned above. They will be eligible if they (i) are aged 16 or older, (ii) are enrolled at one of the participating universities, (iii) have access to a computer, laptop, tablet or mobile device with internet access (iv) score 32 or above on the Irrational Procrastination Scale (IPS) and (v) provide informed consent. We used the cut-off score of 32 as a previous study successfully used it to identify individuals suffering from procrastination (Rozental et al., 2015b). Participants are excluded if they show any suicidal risk by identifying if they have had thoughts of killing themselves or have made plans of killing themselves in the past year and then responding to the question “How likely do you think it is that you will act on this plan in the next 12 months?” with: somewhat likely, likely or very likely. Recruitment started on 19th November 2021.

### 2.3 Procedure

We recruit participants in three ways: First, via the yearly online survey of the WHO WMH-ICS that canvasses the mental well-being of students. All students at the participating universities receive this survey via email. At the end of the survey, students can choose to receive feedback on their mental health, and students who show increased procrastination tendencies (IPS ≥ 32) are invited to sign up for the GetStarted program. Second, marketing activities are conducted online via social media and on-campus via posters, flyers and other advertisements. Third, we recruit participants through the staff who work directly with students (e.g., student psychologists, study advisors and lecturers) by asking them to recommend it to the students who might benefit from the program. Eligible participants receive an email containing the details of the study and an informed consent form. Once informed consent is received, students are randomized to either the treatment or waitlist control group. Participants receive no compensation for completing the program. However, all participants who complete the screening, baseline and post-test questionnaires receive a shopping voucher of 10 euros for an e-commerce platform.

### 2.4 Randomization

An independent researcher who is not involved in the study generates the random sequence using a computer random sequence generator. Randomization is at an individual level, stratified by university site and gender. Participants are randomized into two groups (web-based intervention vs WL) with an allocation ratio of 1:1. We conduct block randomization with randomly varied block sizes (6 to 12 allocations per block) to prevent foreknowledge of intervention assignment. The allocation is concealed to the study's researchers. However, it is not possible to mask personnel and participants to the treatment allocation because of the nature of the intervention.

### 2.5 Intervention

*GetStarted* uses the evidence-based principles of cognitive-behavioral- therapy (CBT). It comprises five main modules and four optional modules that are all delivered via computer, laptop, tablet, or mobile phone (any device that allows internet access). The five main modules focus on the core mechanisms underlying procrastination that are relevant to all those struggling with procrastination. These modules employ CBT techniques and cover the following topics: The first module, through psychoeducation, offers an introduction to the process of procrastinating and an overview of the *GetStarted* program. It closes with a journaling assignment to help students identify procrastination behaviors associated with completing a current task. The second module, through psychoeducation and a journaling exercise, helps students understand the psychological mechanisms underlying procrastination and to recognize the positive and negative nature of procrastination. The third module focuses on unravelling cognitive distortions through exercises which allow students to identify the negative thoughts and feelings that accompany specific tasks, causing them to procrastinate. The fourth module helps students replace these unhelpful thoughts with helpful ones through a cognitive restructuring exercise in which they challenge their negative thinking patterns. This module also includes elements of behavioral activation where the student is encouraged to practice the process of ‘starting’ a task. The fifth and final main module encompasses relapses prevention where the student reflects on their progress, prepares for the future through goal setting techniques, and includes a summary of all the modules. Each module takes approximately 30–40 min, and students are advised to complete one module per week.

In addition to the main modules, students can choose to do all, any, or none of the four optional modules. These modules were made optional as they hold practical tools that may not be equally relevant to all students. Through behavioral activation and problem-solving exercises, the modules cover the following topics 1) breaking a big task into smaller parts, 2) creating and sticking to planning, 3) motivational techniques, and 4) increased productivity. All optional modules become available upon completion of the second main module. All nine modules consist of psychoeducation, reflection questions, interactive exercises, and homework assignments. The content is delivered textually and visually, including pictures, infographics and videos. The intervention is available to the participants on the caring universities platform (caringuniversities.com), which they can access anytime with their username and password. The duration of the program will be between approximately four and eight weeks. However, participants can follow the program at their own pace. Additionally, they are given the option to participate in the program anonymously.

#### 2.5.1 Guidance

During the registration process, participants select an e-coach to support them during the program. The e-coach provides asynchronous, textual feedback via the platform within 72 h (counting workdays only) after every module completed by the participant. The aim is to increase motivation and adherence. E-coaches are (research) master students in clinical psychology and 3rd-year clinical psychology bachelor students (at the end of the 2nd semester) with specific knowledge of low-intensity treatment theory and skills. These coaches complete 6 h of training on their role as an e-coach, written language analyses, how to determine core messages, motivational interviewing techniques, good structure for feedback, crisis management, protocol adherence and several practice sessions before starting their coaching activities. Apart from this, they are required to attend weekly one-hour intervision meetings, which are supervised by the research team. Participants also receive automatic email reminders if they are inactive for 2 weeks without having finished the program.

### 2.6 Control condition

Participants assigned to the wait list control group will receive no intervention for 4 weeks post randomization. During this time, they will be free to utilize any available usual treatment options such as GP's, student psychologists and academic counsellors.

### 2.7 Assessments and outcomes

All assessments take place via the Caring Universities platform. At each time point, the participants receive a link to the assessment questionnaire via email. They get reminder emails to fill out the questionnaire 1 and 2 weeks after receiving the questionnaire. If they still don't fill it out, they will be called within a few days to remind them about the questionnaire. The reminder calls are made by the e-coaches, except for the anonymous participants who will be called by a researcher in the study. All outcomes and their time point measures are listed in Table 1.

**Table 1:** Time point measures of outcome assessments.

Outcome	Measure	Time of assessment					
		T0	T1	T2	T3	T4	T5
Socio-demographics	SR	X					
Procrastination	IPS	X		X	X	X	X
Depressive symptoms	PHQ-9		X	X	X	X	X
Stress	PSS		X	X	X	X	X
Anxiety	GAD-7		X	X	X	X	X
Quality of life	MHQoL		X	X	X	X	X
Satisfaction with treatment	CSQ-8			X		X	
Program usability	SUS-10			X		X	
Satisfaction with e-coach	WAI-I			X		X	
Treatment adherence	Number of modules completed		X	X	X	X	

Note: Abbreviations (alphabetical): CSQ-8: Client Satisfaction Questionnaire – 8 item version; GAD-7: Generalized Anxiety Disorder scale – 7 item version; IPS: Irrational Procrastination Scale; MHQoL: Mental Health Quality of Life questionnaire; PHQ-9: Patient health Questionnaire – 9 item version; PSS: Perceived Stress Scale; SR – self report measure; SUS-10: System Usability Scale – 10 item version; T0: eligibility screening – both groups; T1: baseline screening – both groups; T2: 4 weeks post baseline – intervention group; T3: 4 weeks post baseline – waitlist group; T4: 8 weeks post baseline – waitlist group; T5: 6 months post baseline – both groups; WAI—I: Working Alliance Inventory for guided internet interventions.

#### 2.7.1 Primary outcome – procrastination tendencies

*The Irrational Procrastination Scale* (IPS) is used to assess procrastination tendencies by assessing the extent to which participants procrastinate (Steel, 2007, Steel, 2010). This scale contains nine items scored on a five-point Likert scale ranging from 1 (very seldom or not true to me) to 5 (very often true or true to me). An example of an item is “My life would be better if I did some activities or tasks earlier”. The total scores can range from 9 to 45, with a higher score indicating higher levels of procrastination. With a good internal consistency of α = 0.91 (Steel, 2010), test-retest reliability of *r* = 0.84 (Guilera et al., 2018; Rozental et al., 2014), the IPS has also shown good content, structural, and substantive validity (Shaw and Zhang, 2021).

#### 2.7.2 Secondary outcomes

The secondary outcomes are symptoms of depression, stress, anxiety and quality of life.

*The Patient Health Questionnaire* (*PHQ-9*) is used as the measure of depression (Kroenke and Spitzer, 2002). This questionnaire consists of nine items that can be answered on a four-point Likert scale ranging from 0 (*not at all*) to 3 (*nearly every day*). The total scores can range from 0 to 27, reflecting the severity of depression. Therefore, the higher the score, the more severe the symptoms of depression. The PHQ-9 has been shown to have high sensitivity (0.71–0.84), specificity (0.90–0.97), internal consistency (Cronbach's α = 0.86–0.89), test-retest reliability (*r* = 0.84) and validity (Kroenke et al., 2010; Wittkampf et al., 2007).

*The Generalized Anxiety Disorder scale* (*GAD-7*) is used to measure symptoms of generalized anxiety (Spitzer et al., 2006). This 7-item scale reflects the principal features of GAD such as worrying too much, feeling nervous or on the edge about different things. All items are measured on a four-point Likert ranging from 0 (*not at all*) to 3 (*nearly every day*). The total scores can range from 0 to 21 with higher scores indicating more severe GAD symptoms. The GAD-7 is shown to have high sensitivity (0.89), specificity (0.82), internal consistency (Cronbach's α ≥ 0.85), test-retest reliability (*r* = 0.84), validity, and is recommended as a valid screening tool for assessing the severity of GAD in clinical practice and research (Spitzer et al., 2006; Löwe et al., 2008; Rutter and Brown, 2017).

*The perceived stress scale* (*PSS-10*) is used as a self-report measure of perceived stress (Cohen et al., 1983). It consists of ten items measured on a five-point Likert scale ranging from 0 (*never*) to 4 (*very often*) and it reflects the degree to which the individual had perceived life as unpredictable, uncontrollable, and overloading in the past month. The total scores range from 0 to 40 with a higher score indicating higher levels of perceived stress. The PSS-10 is shown to have high validity, internal consistency (Cronbach's α = 0.74–0.91) and test-retest reliability (*r* = 0.74–0.88) (Cohen et al., 1983; Lee, 2012).

*The Mental Health Quality of Life questionnaire* (*MHQoL*) is used to measure quality of life (van Krugten et al., 2022). It consists of 7 items covering the dimensions of self-image, independence, mood, daily activity, physical health, relationships and future. Each item is measured on a four-point Likert scale ranging from 0 (*very dissatisfied*) to 3 (*very satisfied*). The total scores can range from 0 to 21 with higher scores indicating better quality of life. The MHQoL has been shown to have good psychometric properties in terms of validity, test-retest reliability (*r* = 0.85) and internal consistency (Cronbach's α = 0.85) (van Krugten et al., 2022).

#### 2.7.3 Moderators and covariates

Previous studies have indicated that internet-based interventions rarely have a universal effect on all participants (Ebert et al., 2013; Karyotaki et al., 2018; Reins et al., 2021). Based on this, several socio-demographic variables and other program related variables will be assessed as potential covariates and moderators.

*Sociodemographic information* specifically age, gender, ethnicity, marital status, and whether the participant is currently receiving any psychotherapy or medication will be assessed as potential covariates.

*The Client Satisfaction Questionnaire* (*CSQ-8*) (Larsen et al., 1979) is used to measure the effects of participants' satisfaction with the overall intervention on the treatment outcome. The scale consists of eight items on a four-point Likert scale ranging from 1 (*low satisfaction*) to 4 (*high satisfaction*). The total scores can range from 8 to 32, with higher scores indicating greater satisfaction. The CSQ-8 has shown high reliability and validity for web-based interventions (Boß et al., 2016).

A single additional item is used to measure the participants' satisfaction with the individual modules of the intervention. At the end of each module the participants respond to the question “How useful was this module?” on a scale from 0 to 100, with higher scores indicating greater satisfaction with the module.

*System Usability Scale* (*SUS-10*) (Brooke, 1996) is used to measure the effects of program usability on treatment outcomes. It consists of ten items on a five-point Likert scale from 1 (*strongly disagree*) to 5 (*strongly agree*). The total scores range from 0 to 100 with higher scores indicating higher usability. The SUS-10 has become the most widely used standardized questionnaire to assess perceived usability and has shown good psychometric properties (reliability and validity) (Lewis, 2018).

*Adherence*, defined as “the degree to which the user followed the program as it was designed” (Donkin et al., 2011) is measured by dividing the number of main modules completed by a participant at time of post-test by the total number of main modules in the program, and multiplying this by 100 (Etzelmueller et al., 2020). The resulting percentage will indicate completion rate, with a higher value signifying greater treatment adherence.

*The Working Alliance Inventory for guided internet interventions* (*WAI—I*) (Gómez Penedo et al., 2020) is used to evaluate the effects of participant's satisfaction with the e-coach on treatment outcomes. The WAI-I consists of twelve items on a five-point Likert scale with a total score ranging from 12 to 60, where higher scores indicate higher satisfaction. The psychometric characteristics of the WAI-I yielded adequate results (Gómez Penedo et al., 2020).

### 2.8 Sample size estimation

The power calculation is based on head-to-head comparison of the guided web-based intervention versus the waiting list (*t*-test). We anticipate a conservative estimate of Cohen's d = 0.50 based on a recent meta-analysis of randomized controlled trials that indicated an effect size of 0.55 for cognitive behavioral treatments for procrastination (Rozental et al., 2018b). Thus, if we set the statistical power at 0.8 and alpha at 0.05, according to two tails hypothesis, we need 63 participants per group (total *n* = 126) to detect an effect size of Cohen's d = 0.50. Previous literature has shown that guided web-based interventions have a dropout rate of 28 % (Richards and Richardson, 2012). Thus, considering the potential dropouts, the minimum required sample for the RCT is 176 participants with a target of 88 participants in each arm.

### 2.9 Data analyses

Data on the primary endpoints and the majority of data on the secondary endpoints are continuous. All randomized participants will be included in the analyses based on the intent to treat principle (ITT). Missing values will be imputed using multiple imputations. In addition, we will conduct per protocol analysis, including only those who completed post-treatment and follow-up assessments. All analyses will be conducted using SPSS 26.

#### 2.9.1 Primary outcome

Change in procrastination scores on the IPS will be assessed at post-treatment and follow-up assessments using multilevel mixed models linear regression with a restricted maximum likelihood algorithm for normally distributed data. Binomial models will be used for skewed data. The post-treatment score will be used as the dependent variable and trial arm condition (web-based transdiagnostic intervention vs WL) as independent while adjusting for baseline IPS scores. We will calculate the effect size of Cohen's d by subtracting the intervention group's average score on IPS measures from the average scores of the control group at the post- treatment and dividing the results by the pooled standard deviations. In addition, we will calculate the means and standard deviations of the groups at all time points. Analogously, the Cohen's d will be calculated for the difference score between the post-test measurement (t3 for the intervention group and t4 for the wait list group) and follow-up assessment (6 months, t5).

The reliability change index (RCI) will determine clinically significant change (Jacobson and Truax, 1991). The RCI will be calculated using the standard deviations at baseline and the test-retest reliability coefficient of the IPS (Guilera et al., 2018; Rozental et al., 2014). Participants will be classified as improved, deteriorated or no change based on the RCI for pretest and posttest measures.

#### 2.9.2 Secondary outcomes

For quality of life (MHQoL), depressive symptoms (PHQ-9), symptoms of anxiety (GAD-7) and stress-related symptoms (PSS-10), we will follow the same statistical approach described above for the IPS (with means, standard deviations and Cohen's d). Effects of the intervention on depression and anxiety will be analyzed separately for students scoring above the PHQ-9 (10 or more points) and GAD-7 (10 or more points) at baseline.

#### 2.9.3 Exploratory analyses – covariates and moderators

Descriptive statistics will be used to describe the socio-demographic characteristics of the participants in terms of age, gender, ethnicity, marital status, and whether the participant is currently receiving any psychotherapy or medication. Descriptive statistics will also be used for data exploration on the outcomes of the CSQ-8, SUS, WAI-I and adherence. Additionally, these outcomes will be tested as predictors and moderators or treatment outcome. The moderation analyses will be conducted using PROCESS macro for SPSS version 3.4. The macro provides bias-corrected 95 % confidence intervals (CI) for the indices using a bootstrap calculation based on 5000 samples (Hayes, 2017).

## 3 Discussion

To the best of our knowledge, this is the first randomized controlled trial to examine the effectiveness of an internet-based intervention guided by trained clinical psychology student e-coaches for procrastination in a student population. In addition, this study will examine whether the treatment has any effect on secondary outcomes such as depression, anxiety, stress, and quality of life. We anticipate that the program will be effective in reducing procrastination behaviors. We also expect improvement in depressive symptoms, anxiety, stress and quality of life as they are closely associated with procrastination (Sirois, 2007; Flett et al., 2016; Stead et al., 2010; Beutel et al., 2016).

The results from this high-quality and adequately powered trial will add to the scarce evidence on the effectiveness of a guided internet-based intervention for treating procrastination in university students. We believe this study could provide a flexible and low-intensity treatment for procrastination and prevent common mental health problems in students. A recent meta-analysis on e-health interventions showed that guidance by non-clinicians can be equally effective compared to guidance delivered by specialists and clinicians (Leung et al., 2022). Therefore, using trained psychology students as e-coaches provides a cost-effective and scalable treatment method. In addition, we believe that the systematic screening process, personalized feedback, and a platform to communicate with an e-coach are promising approaches to allow students to recognize a problem and facilitate treatment seeking behaviors (Czyz et al., 2013).

The current study also assess the participants' satisfaction with treatment and e-coaches, program usability and treatment adherence. This will enable us to improve our program and the treatment experience further. Furthermore, the potential moderating variables will give us preliminary insights into who would benefit most from the treatment. Finally, the effectiveness of the intervention is tested in the same setting as it will be implemented. Moreover, while the participants receive compensation for completing all questionnaires, they do not receive any compensation for completing the program. This allows us to examine the real-life efficacy, demand and appeal of *GetStarted*.

The study's results should be considered in light of the following limitation. First, it should be noted that participation in the trial was completely voluntary. Therefore, it could be possible that participants differed from non-participants on important characteristics such as pretreatment motivation and help seeking behaviors that are known to influence treatment outcomes (Patterson et al., 2019; Sansfaçon et al., 2020). Additionally, it could be possible that severe procrastinators “put off” their participation long enough to miss out on treatment entirely. Nevertheless, our study was adequately powered to address the research question and the participants are likely to be a good representation of students that are open to e-health interventions. Second, given the nature of procrastination, it is possible that the study could encounter several dropouts and treatment adherence issues (Rozental et al., 2015a). Therefore, participants will receive support from e-coaches to increase motivation and adherence. Third, while the comparison with an inactive control precisely distinguishes the impact of the intervention, it can also overestimate the effects of treatment (Furukawa et al., 2014). In this study, participants in both groups will have access to treatment-as-usual options. Therefore, the results can be attributed to the actual effectiveness of the program. Fourth, we will not be able to make long-term comparisons between treatment and WLC as the control group will have access to the program four weeks post-randomization. However, the 6-month follow-up will allow a long-term within-group comparison for sustained treatment effectiveness. Finally, we use self-report measures that reflect the self-assessments of actual behaviors but could also be contaminated by other factors such as willingness to report difficulties and overestimating difficulties (Steel et al., 2001). Moreover, we use a non-validated single-item measure to assess participants' satisfaction with each module. However, previous studies have demonstrated that single-item measures can be practical alternatives to multi-item scales (van Hooft, 2014).

A natural continuation of the study would be to compare *GetStarted* with an active control to confirm the study's results and to expand evidence on the effectiveness of this program. Furthermore, the addition of ecological momentary assessments can be beneficial in determining the chronology of change. Finally, the recruitment of participants with procrastination and comorbid depression or anxiety (as established by a diagnostic tool) could evaluate and confirm the transdiagnostic treatment potential of the program.

## 4 Conclusion

This protocol describes the design of a randomized controlled trial to evaluate the effectiveness of a guided internet-based intervention (*GetStarted*) for treating procrastination. The results from this study can build evidence for the effectiveness of a guided internet-based intervention for treating procrastination in university students.

Should it be effective, *GetStarted* could provide a flexible, low intense and cost-effective treatment for procrastination and for the prevention of common mental health problems.

## Declaration of competing interest

The authors declare that they have no known competing financial interests or personal relationships that could have appeared to influence the work reported in this paper.
